# Short-Term and Long-Term Effects of an Exercise-Based Patient Education Programme in People with Multiple Sclerosis: A Pilot Study

**DOI:** 10.1155/2017/2826532

**Published:** 2017-08-16

**Authors:** Christina Lutz, Stephanie Kersten, Christian T. Haas

**Affiliations:** ^1^Sports Science Institute, Saarland University, Campus, 66123 Saarbrücken, Germany; ^2^Faculty of Health Science, LUNEX-International University of Health, Exercise and Sports, 50 Avenue du Parc des Sports, 4671 Differdange, Luxembourg; ^3^Faculty of Health & Social Sciences, Institute for Complex Health Research, Hochschule Fresenius, University of Applied Sciences, Limburger Straße 2, 65510 Idstein, Germany

## Abstract

*Background. *Although people with Multiple Sclerosis (pwMS) benefit from physical exercise, they still show reduced physical activity and exercise behaviour. This study aimed to investigate short- and long-term effects of an exercise-based patient education programme (ePEP) that focuses on empowering pwMS to a sustainable and self-regulated exercise training management.* Methods.* Fourteen pwMS were randomly assigned to immediate experimental group (EG-I: *n* = 8) and waitlist-control group (EG-W: *n* = 6) and attended biweekly in a six-week ePEP. All participants were measured for walking ability, quality of life, fatigue, and self-efficacy towards physical exercise before and after the ePEP, after 12 weeks, and one year after baseline. Short-term effects were analysed in a randomised control trial and long-term effects of all ePEP participants (EG-I + EG-W = EG-all) in a quasi-experimental design.* Results. *Only functional gait significantly improved in EG-I compared to EG-W (*p* = 0.008, *r* = −0.67). Moderate to large effects were found in EG-all for walking ability. Not significant, however, relevant changes were detected for quality of life and fatigue. Self-efficacy showed no changes.* Conclusion.* The ePEP seems to be a feasible option to empower pwMS to a self-regulated and sustainable exercise training management shown in long-term walking improvements.

## 1. Introduction

Numerous studies have demonstrated positive effects of physical exercise in walking ability, fatigue, and quality of life (QoL) in people with Multiple Sclerosis (pwMS) [1–6]. However, pwMS still show reduced physical activity and exercise behaviour compared to healthy people [7–9]. Until now, most of the existing exercise programmes for pwMS are fully supervised and contain a strict training protocol [1]. These programmes rarely analyse long-term effects of physical exercise or focus on the patients' sustainable independence in training management beyond the exercise programme.

Multiple Sclerosis (MS) is a very complex disease and characterised by different disease courses, various symptoms, and an unforeseen disease progress [10–13]. Diurnal and seasonal changes of disease-related symptoms and conditions require daily disease coping and management. Thus, a predefined and strict exercise programme is hard to implement in patients' everyday life. In order to manage disease and exercise training independently and sustainably, pwMS have to be empowered in MS-specific physical exercises and training management. Patient education programmes (PEPs) pursue a common goal to satisfy these claims of empowerment and are well established in several chronic diseases, as well as in MS [14–16]. However, there is a lack of PEPs that focus on exercise training in MS [16, 17]. The few existing programmes, focusing on physical exercise and activity, are mainly Internet-based interventions containing only one or two types of training (cardiorespiratory and/or strength training) with heterogeneous programme goals based on different theoretical concepts [17]. The German research group around Tallner [18] evaluated an Internet-based physical activity promotion programme (MS-Intact) that provides a three-month individualised exercise training programme focusing mainly on endurance and strength training as well as general physical activity promotion based on Social Cognitive Theory (SCT). Results of this randomised control trial (RCT) showed that the Internet intervention had positive effects on muscle strength, lung function, and physical activity; however there were no effects on QoL, fatigue, or aerobic capacity. The research group around Motl [19] and Dlugonski [20, 21] evaluated in three studies a three-month Internet intervention based on SCT with the aim of increasing physical activity in pwMS. The intervention did not focus on specific types of exercise training. Results of these three studies showed an increase of physical activity measured by self-reported questionnaires [19] as well as objective measurements [20, 21] and an increase in exercise and physical activity goal setting. A third research group [22] evaluated a face-to-face intervention for increasing self-regulated exercise behaviour and improving important health outcomes in pwMS. The Transtheoretical Model was used as a guiding framework to promote exercise behavioural change. Exercise training comprised mainly aerobic and strength training and to a lesser extent, balance and stretching exercises. The intervention included supervised and home-based exercise sessions with a decrease in supervised sessions over time. Participants increased self-reported exercise volume and improved fatigue and many QoL domains after three months. The improvements in emotional well-being, social function, and overall QoL were maintained for nine months. Although all named studies could indicate some positive effects on physical activity, exercise behaviour, and physical and psychometric conditions, these programmes did not offer a comprehensive exercise-based patient education programme including essential theoretical and practical knowledge of all exercise training types (endurance, strength, coordination, and stretching) that will enable participants to choose and perform a self-regulated, goal-directed, but also motivating, exercise training.

To our knowledge, the pilot study by Kersten et al. [23] is the first that evaluated a comprehensive exercise-based PEP. This programme was designed as a 12-week face-to-face group intervention aiming at long-term self-regulated exercise training for pwMS. Participants significantly improved in walking distance, gait velocity, TUG, fatigue, and QoL after 12 weeks and maintained these improvements in TUG and gait velocity one year after baseline. Qualitative data indicate improvements in self-confidence and the ability of identifying training strategies and barriers. These positive results show that an exercise-based patient education programme is a feasible option: the programme provides salient knowledge about basic physiological functions and training principles and helps patients to apply this knowledge successfully to the exercise training management [23]. Based on these experiences, a revised exercise-based patient education programme (ePEP) with related contents in a more compact form was developed. The aim of this study is to evaluate the effects of the revised six-week ePEP on self-regulated and long-term exercise behaviour. In contrast to Kersten and colleagues [23], RCT was used to evaluate the immediate effects of the revised ePEP. We hypothesised that teaching pwMS the theoretical and practical essentials of exercising with MS disease, as well as teaching them training- and self-management skills, will enable them to work out independently and sustainably beyond the education phase measured by changes in walking ability, QoL, fatigue, and self-efficacy. Therefore, three working hypotheses were formulated for the different analytic steps.

**Hypothesis 1 .**

A six-week ePEP leads to significant differences in walking ability, QoL, fatigue, and* self-efficacy towards physical exercise* in EG-I compared to EG-W.

**Hypothesis 2 .**

A six-week ePEP leads to significant differences in walking ability, QoL, fatigue, and* self-efficacy towards physical exercise* in pwMS immediately (*T*1/*T*1′) and 12 weeks after the ePEP (*T*2/*T*2′).

**Hypothesis 3 .**

PwMS, who attended a six-week ePEP, show significant differences in walking ability, QoL, fatigue, and* self-efficacy towards physical exercise* one year after baseline measurements (*T*3) compared to baseline (*T*0) due to performing self-regulated physical exercise training beyond the intervention.

## 2. Material and Methods

### 2.1. Study Design and Procedure

**Figure 1:**
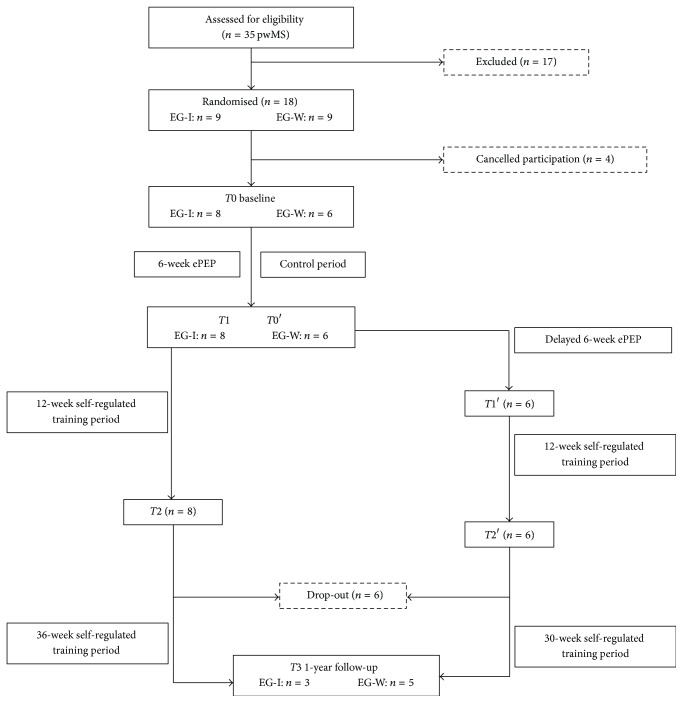
Flow chart of the study design.* Note*. EG-I: immediate ePEP group; EG-W: waitlist-control group;* T*0: baseline measurements;* T*0′: second baseline measurements for EG-W;* T*1/*T*1′: measurements after six-week ePEP;* T*2/*T*2′: measurements after 12-week self-regulated training period;* T*3: measurements one year after baseline.

This long-term pilot study was structured in three parts: (1) RCT analysing the immediate effects of the six-week ePEP with a waitlist-control group; (2) a quasi-experimental design (QED) analysing the short-term effects of all participants who received the ePEP, immediately and 12 weeks after the ePEP; and (3) a one-year follow-up test analysing the long-term effects of all ePEP participants. Please see Figure 1. With the knowledge of beneficial effects of exercise training on health conditions in pwMS, the control period of the waitlist-control group should not take too long. Therefore, the RCT was only proposed for the six-week ePEP. Merging the data of both exercise groups in the QED allows the analysis of a bigger sample size in awareness of possible drop-outs. The study was approved by the local ethics committee of the Hochschule Fresenius, University of Applied Science (Idstein, Germany). Participants were recruited between May and July 2013 by the German Society of Multiple Sclerosis and the health insurance company IKK Südwest and were manually randomly assigned to immediate ePEP group (EG-I) and waitlist-control group (EG-W) by the study board. After baseline measurements (*T*0), EG-I started with the six-week ePEP, whereas the participants of EG-W were instructed not to change their daily routines. After six weeks, EG-I and EG-W were tested again (*T*1 = EG-I/*T*0′ = EG-W). While EG-W received the six-week ePEP, EG-I started a 12-week self-regulated training period. EG-W were tested again after the ePEP (*T*1′) and started the 12-week self-regulated training period afterwards. Both groups were tested after their self-regulated training period (*T*2/*T*2′) and one year after baseline (*T*3). Participants were offered to contact the project board for support on training management.

### 2.2. Participants

Thirty-five pwMS were recruited and screened for eligibility criteria by their neurologists and general practitioners. Inclusion criteria were as follows: (a) definite diagnosis of MS; (b) age ≥ 18; (c) documentation of the current state of disease (EDSS score, medication, and clinical course); (d) disease-related problems in daily life (self-reported); (e) ability to stand and walk with or without assistive devices (self-reported); (f) physician approval for beginning a physical activity programme; (g) signed letter of written informed consent. Exclusion criteria were as follows: (h) MS relapse, changing medication, or cortisone therapy one month prior to recruitment and during the study; (i) concurrent neurological, internal, or orthopaedic disorders interfering with standing and walking ability; (j) participation in other active therapies during the study. Prior sport participation or affinity was neither an exclusion nor an inclusion criterion. The inclusion and exclusion criteria were only applied for the period of the six-week ePEP and the 12-week self-regulated training. Exclusion criterion (h) was also held for the one-year follow-up test. During the 30- and 36-week self-regulated training period, participants were free to make their own decisions in their disease management (medical treatment, diet, etc.) and the implementation of their exercise training. Eighteen out of the 35 pwMS enrolled in the programme remained and were randomly assigned into immediate ePEP group (EG-I: *n* = 9) and waitlist-control group (EG-W: *n* = 9). Due to the study design, there was no possible blinding of the participants and study board. Assistances for measurements were blinded. Participants were given written and oral information about the study in advance.

### 2.3. Intervention

The ePEP was developed based on study results by Kersten et al. [23] to provide pwMS with the knowledge to work out independently. Participants were taught neurophysiological essentials in MS disease, (neuro-) physiological effects of sports, and physical exercises in general and specific for MS, MS-specific recommendations of exercise training [24], training principles, and the importance of resting periods. In order to guarantee a comprehensive treatment, various types of exercise training (cardiorespiratory, strength, coordination/reflex-based, and flexibility) were offered based on individual performance abilities. A main topic of the exercise training was the theory and praxis of highly reflexed-based movements applied as deviance-based gait training (DGT) [25]. There is evidence for an increased production of neurotrophic factors (NF) produced by moderate exercises [26–30] and highly reflex-based movements such as running [31]. These NFs show a beneficial influence on neural function [32] and on relapse remission [33]. In contrast, immobility and forced nonuse may potentiate the neurodegeneration [34]. With respect to existing indications of reduced production of NFs in pwMS [30, 35–37], these neurophysiological contents have been shown to be key aspects in this ePEP and are the theoretical framework for the DGT. The DGT comprises various walking and running exercises such as short sprints, walking alternatively forward, backward, sideward, jumps, and hops, because these are natural (highly) reflex-based movements and lead to an increased NF production compared to swimming or exercises in standing position [31]. Furthermore, the DGT has the aim to vary several movement patterns based on studies of motor learning [38]. These studies recommend variable and random training practice to gain a better performance outcome in retention tests than with constant and blocked training practice. Psychological determinants for adoption and maintenance of health-related behaviour, such as self-efficacy, problem-solving, and patient-generated goal setting were taught in order to enhance patients' exercise motivation and self-management skills [39–41]. Focusing on the sources of self-efficacy, we explained to pwMS the benefits of exercise training (positive outcome expectations) and offered opportunities to experience the four main sources of self-efficacy such as mastery experience, vicarious experience, symbolic experience, and emotional arousal (feedback). Mastery and vicarious experience were made during the practical exercise training in the ePEP and at home. With guidance and support by the sport scientist, group members, friends, or family members, participants gained symbolic experience. Furthermore, external feedback (from the sport scientist, etc.) and internal feedback from the participant (feelings, signs of the body, etc.) will lead to positive emotional arousal. Group discussions, assignments, and the documentation of the training and symptoms were contained in the ePEP to highlight the four experiences and to support participants in the acquisition of strategies for problem-solving. Besides, we taught pwMS how to set individual goals using the S.M.A.R.T. concept [41]. The main contents of each lesson are shown in Table 1. The ePEP were delivered over six weeks, twice a week for 60 to 90 minutes per session. The theoretical and practical contents were provided in a distribution of 40 : 60 per lesson with different pedagogical methods to consider physical and mental fatigue and to provide an optimal learning atmosphere. Predefined and self-chosen breaks were undertaken for the physical and mental regeneration. The sessions were supervised by at least one sport scientist and one assistant. Two patient booklets with theoretical background and practical information were provided for the participants to exercise and complete homework beyond the sessions. The participants were encouraged to document their training habits, any symptoms, and daily activities in order to analyse experiences, training progression, or contraindications. After the ePEP, pwMS performed their exercise training with a self-generated training schedule autonomously at home for 12 weeks and a further 30 (EG-W) or 36 (EG-I) weeks until one year after baseline.

### 2.4. Measurements

Walking ability and psychometric outcomes were measured in the EG-I at four test times (*T*0,* T*1,* T*2, and* T*3). The EG-W had one additional measurement after their control period (*T*0′). Please see Figure 1.

#### 2.4.1. Walking Ability

Walking endurance was measured using the Six-Minute-Walk-Test (6MWT) as a valid and reliable measurement to assess functional walking capacity in people with pulmonary diseases as well as in pwMS [42–44]. Participants were instructed to walk as far as possible in six minutes, using their regular assistive devices if needed. Walking speed was assessed by Timed-Up-and-Go-Test (TUG), which is a quick, reliable, and valid test to quantify functional mobility [45, 46]. The fastest trial time out of three was used for further analyses. The Functional Gait Assessment (FGA) is a further development of the Dynamic Gait Index and assesses postural stability during various walking tasks. It is evaluated for neurological diseases (e.g., Parkinson Disease, Strokes) and for older adults [47–51]. The FGA includes ten items, where performance of each item is rated from 0 (severe impairments) to 3 (normal gait), with a maximal FGA sum score of 30 points [47, 48].

#### 2.4.2. Quality of Life

The Multiple Sclerosis International Questionnaire of Quality of Life (MusiQol) is a valid and reliable multidimensional, 31-item questionnaire for assessing disease-specific QoL in pwMS [52, 53]. It has been evaluated for the German language and has been shown to provide validity and reliability [53]. Cronbach's alpha coefficients ranged from 0.68 to 0.92 as reported in the previous study [52]. The global index score is calculated as the mean of the nine individual dimension scores. A higher score indicates a higher QoL [54].

#### 2.4.3. Fatigue

The WEIMuS scale (German: Würzburger Erschöpfungsinventar bei MS) is a two-dimensional questionnaire to detect fatigue (total, mental, and physical) by 17 items and is validated in German pwMS [55]. Reliability analyses in the German evaluation study indicated Cronbach's alpha coefficient of 0.92 [55]. A high sum score indicates increased fatigue.

#### 2.4.4. Self-Efficacy

The SSA scale (German: Selbstwirksamkeit zur sportlichen Aktivität) was used to measure* self-efficacy towards physical exercise* [56]. The SSA scale consists of 12 items; a high sum score indicates a high degree of conviction to execute physical exercises despite barriers. The SSA scale is a valid and reliable measurement of self-efficacy towards physical exercise with a satisfactory internal consistency of alpha = 0.89 as reported from Fuchs and Schwarzer [56].

### 2.5. Statistical Analyses

Only participants who attended a minimum of 80% of all ePEP lessons were considered for statistical analyses. Ordinal or skewed data and the small sample size, resulting from per protocol analysis, required the application of exact nonparametric tests for statistical analyses (SPSS Statistics version 21, IBM, Inc., Chicago, IL). Statistical *p* value was set at *p* ≤ 0.05. Differences in baseline between EG-I and EG-W were analysed by Fisher-Freeman-Halton exact test for categorical variables and by Mann–Whitney *U* test for continuous variables. Analysing the immediate effects of the ePEP in the RCT, Mann–Whitney *U* test (two-tailed) was used to test significant differences between groups on change scores for* T*0 and* T*1 measures. Analysing the immediate effects of the ePEP and the effects after 12-week self-regulated training period of all ePEP participants in the QED, Friedman test was used to assess statistical difference in EG-all (EG-I + EG-W) over time (*T*0/*T*0′,* T*1/*T*1′, and* T*2/*T*2′). In case of significance, post hoc tests (Wilcoxon Signed Rank test) with Bonferroni adjustments were applied. Wilcoxon Signed Rank test (two-tailed) was used to analyse the long-term effects of the ePEP one year after baseline measurements (*T*3) compared to baseline (*T*0/*T*0′).

Effect sizes were calculated using the formula *r* = *z*/square root of *N* (*N* = number of total observations), which is recommended for skewed distributed variables [57]. *Z*-scores were taken from SPSS results of Mann–Whitney *U* test and Wilcoxon Signed Rank test. Effects for *r* are classified into small (*r* = 0.10), moderate (*r* = 0.30), and large (*r* = 0.50) effects [58].

## 3. Results

### 3.1. Baseline

For the RCT, participants were randomly assigned into EG-I (*n* = 9) and EG-W (*n* = 9). Due to cancellation of four participants prior to the start of the study, only eight participants remained for EG-I and six participants for EG-W with no significant differences in sample characteristics and baseline measurements between the groups. Please see Table 2. EG-I consisted of seven women and one man with a mean age of 52.4 (SD ± 10.4), a median EDSS score of 3.5 (IQR: 2.25–3.5), and a mean disease duration of 12.5 (SD ± 10.0) years. EG-W, only women, had a mean age of 56.0 (SD ± 7.4), a median EDSS score of 3.5 (IQR: 2.0–3.5), and a mean disease duration of 17.5 (SD ± 7.4). For the evaluation of the QED, outcomes of both groups (EG-I/EG-W) were summarised in EG-all for* T*0/*T*0′,* T*1/*T*1′,* T*2/*T*2′, and* T*3. Six participants were lost to the one-year follow-up test (holidays: *n* = 2, illness: *n* = 2, and no answer: *n* = 2). The compliance during the six-week ePEP was high with an attendance over 80% for all participants (= not more than two missing lessons). No adverse events were reported during the study.

### 3.2. Immediate Effects of Intervention after Six-Week ePEP (RCT)

Mann–Whitney *U* test on change scores showed a significant difference and a large effect between the groups for FGA (*Z* = −2.503, *p* = 0.008, and *r* = −0.67) only. No statistical differences were detected for TUG, 6MWT, MusiQol, WEIMuS (total, mental, and physical fatigue), and SSA. Nevertheless, MusiQol (*Z* = −1.291, *r* = −0.35), physical fatigue (*Z* = −0.523, *r* = −0.14), and TUG (*Z* = −1.033, *r* = −0.28) showed small to moderate effect sizes and increased mean values in EG-I compared to EG-W. Please see Table 3.

### 3.3. Short-Term Effects of Intervention after Six-Week ePEP and after 12-Week Self-Regulated Training Period (QED)

Significant changes over time (*T*0/*T*0′,* T*1/*T*1′, and* T*2/*T*2′) were found for EG-all in TUG (*χ*^*2*^ = 10.29, *p* = 0.004), 6MWT (*χ*^*2*^ = 10.58, *p* = 0.005), and FGA (*χ*^*2*^ = 17.08, *p* ≤ 0.001) (Table 4). Post hoc analyses showed significant improvements with moderate to large effect sizes from* T*0/*T*0′ to* T*1/*T*1′ for FGA (*Z* = −3.20, *p* = 0.001, and *r* = −0.60) and for TUG (*Z* = −2.45, *p* = 0.033, and *r* = −0.46) as well as significant improvements from* T*0/*T*0′ to* T*2/*T*2′ for TUG (*Z* = −2.76, *p* = 0.010, and *r* = −0.52), 6MWT (*Z* = −2.79, *p* = 0.009, and *r* = −0.53), and FGA (*Z* = −2.59, *p* = 0.018, and *r* = −0.49). There were no significant differences for MusiQol, WEIMuS (total, mental, and physical fatigue), and SSA. However, small to moderate effect sizes were detected for MusiQol (*T*1/*T*1′: *Z* = −2.23, *r* = −0.42;* T*2/*T*2′:* Z* = −2.35, *r* = −0.44), total fatigue (*T*1/*T*1′: *Z* = −1.26, *r* = −0.24;* T*2/*T*2′: *Z* = −0.74, *r* = −0.14), and physical fatigue (*T*1/*T*1′: *Z* = −1.56, *r* = −0.29;* T*2/*T*2′: *Z* = −1.91, *r* = −0.36) in both times. Mental fatigue and SSA showed no or only small effect sizes. Please see Table 4.

### 3.4. Long-Term Effects of Intervention 30 or 36 Weeks after ePEP (QED)

Due to the loss of six participants in the follow-up test one year after baseline, data from the remaining eight participants were compared to baseline data. This analysis showed significant long-term effects with large effect sizes for TUG (*Z* = −2.38, *p* = 0.016, and *r* = −0.60) only. Small to moderate effect sizes with superior mean values were detected for 6MWT (*Z* = −0.84, *r* = −0.21), FGA (*Z* = −0.84, *r* = −0.21), MusiQol (*Z* = −1.88, *r* = 0.42), total fatigue (*Z* = −1.36, *r* = 0.34), mental fatigue (*Z* = −1.36, *r* = −0.34), and physical fatigue (*Z* = −1.05, *r* = −0.26). SSA decreased with a moderate effect (*Z* = −1.18, *r* = −0.3). Please see Table 5.

## 4. Discussion

This exercise-based patient education programme was developed to integrate a goal-oriented, effective, and self-regulated exercise training management in the daily life of pwMS. Drawing from a complex adaptive systems theory [59], the present comprehensive approach focused on considering individual and disease-related needs and goals (e.g., abilities and disabilities, everyday challenges, and character profiles) in a face-to-face intervention. A successful implementation of exercise training in patients' daily life should be reflected in improved walking ability, QoL, fatigue, and* self-efficacy towards physical exercise*. The results of the RCT analysis demonstrated significant improvements with large effects in functional gait for the six-week ePEP group compared to the waitlist-control group. Analysis of all ePEP participants in the QED confirmed this improvement for FGA and, in addition, it identified significant improvements with moderate to large effects in walking endurance and in walking speed immediately and 12 weeks after the ePEP. The benefits in walking speed could be maintained one year after the baseline measurements. The positive study results by Kersten et al. [23] for walking ability, due to teaching pwMS essentials of the MS disease, exercises, and training management, have been confirmed in this revised form of ePEP.

Moreover, the benefits of our study are consistent with the results of three meta-analyses concerning the impact of exercise training on walking mobility [60], QoL [61], and fatigue [62]. These meta-analyses reported an overall small effect from exercise training on walking mobility (Hedges' *g* = 0.19) [60] and QoL (Hedges' *g* = 0.23) [61] as well as an overall moderate effect on fatigue (Cohen's *d* = 0.45) [62]. Most of these included studies only analysed short-term effects of exercise training. This study at hand showed similar short-term effects linked with small to large effect sizes for walking ability and QoL and, additionally, moderate to large long-term effects for walking ability, QoL, and fatigue.

Regarding RCT analysis, only functional gait (FGA) improved immediately after the ePEP in EG-I compared to EG-W. The high amount of DGT with its specific training stimulus trained the participants' functional ability to adopt and perform several different walking tasks such as in the FGA. In contrast, walking speed (TUG) and walking endurance (6MWT) did not change significantly in RCT analysis. We assume that the participants' knowledge of being part of an intervention probably caused effects on unspecific walking and psychometric outcomes in the waitlist-control group* (John-Henry-Effect)* [63]. This is also confirmed by unpublished qualitative data of the study. Participants of the waitlist-control group reported in semi-structured interviews that they were motivated to be more active in their daily lives after the baseline test although they were instructed not to change their daily routines. The beneficial change in FGA and TUG in QED analysis is attributable to neuronal and functional adaptations, whereas the steady-state of walking endurance from* T*0/*T*0′ to* T*1/*T*1′ and the delayed significant increase from* T*0/*T*0′ to* T*2/*T*2′ is probably caused by delayed aerobic and morphologic adaptations. Information from qualitative data indicated that during the 12-week self-regulated training period most of the participants focused their exercise training more on endurance than on walking speed or functional gait. The participants reported that they had implemented exercise training in their daily lives; however, they performed exercises less than intended by their self-generated training schedule.

Only a few studies have examined long-term effects of exercise training. For example, Schwartz et al. [64] evaluated both short- and long-term effects of a robot-assisted gait training (RAGT) compared to conventional gait training (CGT) in gait parameters. Both groups significantly improved after the four-week intervention; however, only the CGT group showed long-term effects with still higher scores compared to baseline. RAGT, CGT, and the ePEP focused on gait exercises, which lead to short-term effects due to the specific stimulus for gait performance. In contrast, long-term effects on walking ability are caused by the functional gait exercises in the CGT and ePEP and also by the transfer of competences in exercise training and training management in the ePEP. Considering that walking difficulties are the most challenging aspects of having MS [65], improvements or maintenance in walking ability allows patients to participate in daily life, which is a very meaningful aspect of the patients' QoL.

Relevant nonsignificant short-term and long-term improvements with moderate effect sizes were detected for QoL and fatigue. Comparing the baseline MusiQol score (mean ± SD 68.1 ± 13.9) with the MusiQol score from an international control sample of 1,992 pwMS (mean ± SD 65.82 ± 14.75) [52], the ePEP sample showed already higher baseline scores with further improvements after the PEP. Although these improvements failed statistical significance, the index score increased about 7.9 points with a moderate effect. This reveals a relevant benefit regarding that pwMS with a mild clinical global impression of severity (CGI) differ in the MusiQol score about 10 points from pwMS with a severe CGI [52].

No significant differences were detected for fatigue scores between EG-I and EG-W after the six-week ePEP. Although total fatigue improved in both groups, Table 4 shows that the EG-I mainly improved in physical fatigue whereas the EG-W improved in mental fatigue. This might be an indication of a positive reaction to the physical stimulus in the ePEP [66]. Analyses of EG-all indicated a decrease in fatigue symptoms immediately after the ePEP with a small effect, a slight increase after the 12-week self-regulated training period, and a further decrease with a moderate effect one year after baseline. In this study, we did not differentiate between fatigue and nonfatigue participants in the analyses. The inclusion of both fatigue and nonfatigue participants in analyses might have impeded significant results and is also known as a predicted reason for heterogeneous findings of exercise training on fatigue in literature [67].

*Self-efficacy towards physical exercise* improved neither in RCT nor in quasi-experimental analyses, although methods based on psychological theories for enhancing self-efficacy [40] were applied in the programme. These results are similar to the results of the Internet intervention for increasing physical activity by Motl et al. [19], which were based on SCT for increasing self-efficacy. Noticeably, baseline scores of SSA in both groups (mean ± SD EG-I: 4.7 ± 0.7; EG-W: 4.4 ± 1.3) and EG-all (mean ± SD 4.7 ± 1.2) are higher compared to scores of norm population of the same age (mean ± SD 3.52 ± 1.5) [56]. Based on findings from unpublished qualitative data (semi-structured interviews), a possible reason for the high SSA score in this study might be the participation of primarily patients with a high affinity to sporting activities. This raises the question, if there is even a further increase possible.

Due to the small sample size, the terminated control group, and a possible clustering, the results of this pilot study allow a limited interpretation. However, the face-to-face education concept of ePEP required a small sample size to guarantee individual guiding and counselling. A multicentre study with a larger sample size is needed to confirm the results. A larger sample size would also allow a more balanced distribution of the gender and a multivariate analysis of confounding effects such as gender, EDSS score, and type of MS. A limitation of this study is that there was no balancing of gender in the sample size and no multivariate analysis to consider confounding effects. The voluntary participation at the ePEP, the fixed starting point of the study, and the higher prevalence of MS in women than in men (ratio 2.3–3.5 : 1) [68] might be reasons for the nonbalanced distribution of gender in the sample size. Furthermore, the results of the QED analysis only allow a limited interpretation due to the fact that a control group is missing and that data from two exercise groups (EG-I + EG-W after their control period) were merged. This was necessary to detect effects of the ePEP in a more meaningful sample size, immediately after the ePEP, after the 12-week self-regulated training period, and one year after baseline. Resulting from data merging of the two groups, there is a time-shift of six weeks within the participants at the one-year follow-up test (EG-W = 30 weeks and EG-I = 36 weeks). Nevertheless, we set a fixed date for the start and end of the study with the assumption that six weeks—more or less—over the span of one year will not influence the results in a significant amount. The negligence of possible occurring relapses and changes in medical treatment during the 30- and 36-week self-regulated training period might have influenced the outcomes at the one-year follow-up test. We are aware of these influences; however, we did not want to intervene in patients' medical treatment decisions and we did not want to exclude participants from analysis that had relapses. The occurrence of relapses is part of the nature of the disease and should not be excluded in the analysis of long-term effects. Furthermore, there are multiple factors in patients' daily life and disease management that we cannot control in a long-term “field study” and that probably have an impact on the outcomes. That is probably the main reason for the lack of long-term studies in MS (and exercise training). Reducing the impact on the outcomes, occurrences of MS relapse, changing medication, or cortisone therapy one month prior to the follow-up test were applied as exclusion criteria. Last but not least, the participants' knowledge of being part of an intervention probably caused effects on outcomes in waitlist-control group* (John-Henry-Effect)* [63]. An extended control period besides the 12-week self-regulated training period might minimise this effect.

## 5. Conclusion

Although the RCT analysis of the six-week ePEP only showed improvements in functional gait, the results of QED detected significant short- and long-term benefits in walking ability as well as relevant benefits in QoL and fatigue. This suggests a realisation of a sustainable and independent training management by pwMS beyond the programme that have resulted in important benefits in walking ability. This ePEP is a feasible and comprehensive approach with the choice of various effective and evidence-based exercises and sport possibilities according to individual needs and preferences concerning MS-specific challenges in training management. The participants also gained knowledge and skills to train independently, economically, flexibly, and goal-oriented without any appointments, journeys, or any special equipment. Regarding that MS is a chronic and degenerative disease with a progression of disabilities, an improvement or nonworsening health condition is a benefit for the patient and its daily life participation. Nevertheless, a further RCT with an optimal sample size should be conducted to confirm the short- and long-term benefits of this ePEP. Providing the contents of ePEP as a weekend workshop could be another possibility of reaching those pwMS who are employed, have families, or live in rural areas.

## Figures and Tables

**Table 1:** Theoretical and practical contents of the ePEP lessons.

Lesson	Theoretical contents	Practical contents
(1) Essentials of MS, physical exercises, and sports	(i) Neurophysiological basics of MS (ii) Effects of physical exercises and sport in general and specifically for MS	(i) DGT

(2) MS-specific physical exercises and sports	(i) Reflex-based exercises and activities (ii) Neurotrophic factors and MS	(i) Warm-up: DGT (ii) Main programme: aerobics/dancing

(3) Coordination training	(i) Essentials^*∗*^ of coordination training (ii) Essentials^*∗*^ of DGT	(i) Warm-up: coordinative exercises and games with balls (ii) Main programme: DGT (patient-led)

(4) Self-management	(i) Comfort and learning zone (ii) Self-efficacy	(i) Warm-up: game with coordinative exercises (patient-led) (ii) Main programme: balance training

(5) Strength training	(i) Neuromuscular basics (ii) Essentials^*∗*^ of strength training	(i) Warm-up: DGT (patient-led) (ii) Main programme: strength training (iii) Cool down: stretching

(6) Training management	(i) Basics of training process (model of super compensation) (ii) Training principles	(i) Warm-up: DGT (ii) Main programme: strength training with latex resistance bands (iii) Cool down: stretching

(7) Cardiorespiratory training	(i) Essentials^*∗*^ of cardiorespiratory training (ii) Determinants in training control (e.g., heart rate measurement, symptoms)	(i) Warm-up: DGT (ii) Main programme: cardiorespiratory training (continuous or interval training) (iii) Cool down: stretching

(8) Cardiorespiratory and flexibility training	(i) Essentials^*∗*^ of flexibility training	(i) Warm-up: game with balls (ii) Main programme: cardiorespiratory training (continuous or interval training) (iii) Cool down: stretching

(9) Motivation and training barriers	(i) Motives, motivation/lack of motivation for physical exercise (ii) Attribution theory (iii) Problem solving skills	(i) Warm-up: DGT (outdoor) (ii) Main programme: strength training (iii)Cool down: stretching

(10) Goal setting	(i) Goal setting: S.M.A.R.T. (ii) Training schedule: principles and instructions (iii) Homework: design individual goals and training schedule	(i) Warm-up: football (ii) Main programme: game with contents of strength and coordinative training

(11) Training schedule and repetition	(i) Discussion of self-designed training schedule; optional consultation	(i) MS Olympics (game consisted of quizzes and exercises)

(12) Final discussion	(i) Discussion of quiz solutions (MS Olympics) and victory ceremony (ii) Instructions and optional consulting for the self-regulated training period (iii) Final feedback	(i) DGT (ii) Final game

*Note*. *∗* included definition, aims, exercises, training methods, training adaption; DGT: deviance-based gait training.

**Table 2:** Sample characteristics.

	EG-I (*n* = 8)	EG-W (*n* = 6)	*p* value
Gender, *n* (male/female)	1/7	0/6	ns
Age (years), mean (±SD)	52.4 (±10.4)	56.0 (±7.4)	ns
EDSS score, median (IQR)	3.5 (2.25–3.5)	3.5 (2.0–3.5)	ns
Time since diagnosis (years), mean ± SD	12.5 (±10.0)	17.2 (±7.4)	ns
Type of MS, *n*			
RRMS/PPMS/SPMS/benign	3/3/1/1	4/1/1/0	ns
Level of education			
Low/middle/high	1/2/5	0/3/3	ns
Medication, *n*			
Immuno/symptom/none	5/2/1	3/1/2	ns
Employment status, *n*			
Employed/unemployed	4/4	4/2	ns
Unemployed due to MS	4	1	—

*Note*. Mean (±SD) and median (IQR: interquartile range 25th–75th) are given for baseline *T*0; *p* values were computed by Fisher-Freeman-Halton exact test for discrete variables and Mann–Whitney *U* test for continuous variables (two-tailed; *p* ≤ 0.05); EG-I: immediate ePEP group; EG-W: waitlist-control group; ns: not significant; EDSS: Expanded Disability Status Scale; immuno: immunotherapy; symptom: symptomatic therapy; low education level: lower secondary school (years 5–9); middle education level: secondary school (years 5–10); high education level: A-level (German university entrance qualification; years 5–13).

**Table 3:** RCT outcomes in walking ability, QoL, fatigue, and self-efficacy for immediate ePEP group and waitlist-control group at baseline (*T*0), after six-week ePEP (*T*1).

Variable	EG-I (*n* = 8)			EG-W (*n* = 6)			Difference between groups	
	*T*0 mean ± SD median (IQR)	*T*1 mean ± SD median (IQR)	Change from *T*0-*T*1 mean (%) median (IQR)	*T*0 mean ± SD median (IQR)	*T*1 mean ± SD median (IQR)	Change from *T*0-*T*1 mean (%) median (IQR)	*Z*-score	Effect size *r*
TUG (s)	7.8 ± 1.8 7.8 (6.2–9.4)	7.2 ± 1.6 7.0 (6.0–8.3)	0.5 (+7.1) 0.2 (−0.0–1.5)	7.8 ± 1.6 6.9 (6.1–8.5)	7.2 ± 1.2 6.7 (5.6–7.7)	0.6 (+8.1) 0.5 (0.3–0.9)	−1.033	−0.28
6MWT (m)	483.2 ± 89.7 488.1 (399.1–541.5)	496.1 ± 128.4 501.0 (396.1–568.3)	−12.9 (+2.7) −21.5 (−38.2–11.6)	526.0 ± 117.5 477.6 (393.4–620.9)	530.5 ± 153.8 532.2 (448.2–639.0)	−4.5 (+0.8) −17.0 (−37.4–34.7)	−0.293	−0.08
FGA (score)	19.5 ± 4.0 20.5 (15.5–21.8)	25.4 ± 3.7 25.5 (23.5–28.8)	−5.9 (+30.1) −6.0 (−8.0–4.0)	20.7 ± 6.6 25.5 (17.8–28.5)	23.3 ± 6.9 28.0 (21.8–30.0)	−2.7 (+12.9) −2.5 (−4.0–−0.8)	−2.503^*∗*^	−0.67
MusiQol (score)	68.1 ± 10.6 69.2 (60.6–75.6)	77.2 ± 11.4 79.5 (66.0–86.2)	−9.1 (+13.4) −9.8 (−15.8–−0.4)	70.0 ± 7.8 74.9 (49.8–83.4)	74.6 ± 11.5 75.1 (62.9–84.4)	−4.6 (+6.5) −5.6 (−9.8–−0.6)	−1.291	−0.35
WEIMuS (score)	26.0 ± 17.7 28.5 (6.5–40.3)	22.1 ± 15.5 22.0 (7.3–34.0)	3.9 (+14.9) 2.0 (−4.0–11.5)	21.3 ± 6.1 15.5 (12.5–29.0)	18.8 ± 9.2 11.5 (9.5–19.3)	2.5 (+11.7) 0.0 (−4.8–9.5)	−0.195	−0.05
Mental fatigue (score)	10.5 ± 10.2 6.0 (1.3–22)	9.5 ± 8.4 9.0 (1.0–16.0)	1.0 (+9.5) 0.0 (−1.0–1.8)	8.8 ± 3.9 7.0 (2.8–10.5)	7.5 ± 2.3 8.0 (5.5–9.3)	1.3 (+15.1) 1.0 (−2.5–5.0)	−0.326	−0.09
Physical fatigue (score)	15.5 ± 10.0 19.0 (4.0–22.8)	12.6 ± 8.3 12.5 (5.8–20.0)	2.9 (+18.5) 1.0 (−3.0–10.3)	12.5 ± 5.8 6.5 (4.5–9.5)	11.3 ± 8.4 7.5 (4.8–22.0)	1.2 (+9.3) −1.5 (−3.5–7.5)	−0.523	−0.14
SSA (score)	4.7 ± 0.7 4.9 (4.4–5.3)	4.2 ± 0.8 4.45 (3.8–4.7)	0.5 (−10.8) 0.2 (−0.1–1.5)	4.4 ± 1.3 4.6 (2.9–6.2)	4.6 ± 1.8 4.5 (3.4–6.4)	−0.2 (+3.4) 0.2 (−0.7–0.4)	−0.646	−0.17

*Note*. Positive percentage values indicate improvements and negative percentage values indicate declines; *Z*-scores were taken from results of Mann–Whitney *U* test; effect size* r* was computed using formula *r* = *z*/square root of *N*; *∗* denotes statistically significant difference between changes of scores of the groups (*p* ≤ 0.05) for exact *p* value of Mann–Whitney *U* test (two-tailed). EG-I: immediate ePEP group; EG-W: waitlist-control group; *T*0: baseline measurements; *T*1: measurements after six-week ePEP; SD: standard deviation; IQR: interquartile range (25th–75th); TUG: Timed-Up-and-Go-Test; 6MWT: Six-Minute-Walk-Test; FGA: Functional Gait Assessment; MusiQol: Multiple Sclerosis International Questionnaire of Quality of Life; WEIMuS: German MS-specific fatigue questionnaire—lower values indicate fatigue improvement; SSA: scale for self-efficacy towards physical exercise.

**Table 4:** Quasi-experimental design outcomes in walking ability, QoL, fatigue, and self-efficacy for EG-all in baseline (*T*0/*T*0′), after the six-week ePEP (*T*1/*T*1′) and after the 12-week self-regulated training period (*T*2/*T*2′).

Variable	EG-all (*n* = 14)					Friedman *T*0-*T*2 *X*^2^*-score*	Post hoc *T*0-*T*1 *Z*-score (effect size *r*)	Post hoc *T*0-*T*2 *Z*-score (effect size *r*)
	*T*0/*T*0′ (*n* = 14) mean ± SD median (IQR)	*T*1/*T*1′ (*n* = 14) mean ± SD median (IQR)	Percentage change *T*0-*T*1	*T*2/*T*2′ (*n* = 14) mean ± SD median (IQR)	Percentage change *T*0-*T*2			
TUG (s)	7.5 ± 1.5 7.2 (6.1–8.8)	7.0 ± 1.4 7.0 (5.7–7.7)	+7.2	6.7 ± 1.1 6.8 (5.8–7.6)	+10.1	10.29^*∗*^	−2.45^*∗*^ (−0.46)	−2.76^*∗*^ (0.52)
6MWT (m)	494.3 ± 113.3 488.1 (398.4–556.2)	516.5 ± 121.1 506.2 (424.3–589.0)	+4.5	539.0 ± 98.4 543.5 (483.3–598.5)	+9.2	10.58^*∗*^	−1.60 (−0.30)	−2.79^*∗*^ (−0.53)
FGA (score)	21.1 ± 5.6 21.0 (16.5–26.0)	25.9 ± 3.7 27.0 (22.8–29.0)	+22.3	24.9 ± 4.3 25.5 (22.0–29.0)	+18.2	17.08^*∗*^	−3.20^*∗*^ (−0.60)	−2.59^*∗*^ (−0.49)
MusiQol (score)	68.1 ± 13.9 69.2 (58.7–82.6)	76.0 ± 11.1 77.5 (64.9–84.4)	+1.6	74.7 ± 15.0 73.9 (59.5–90.3)	+9.6	2.71	−2.23 (−0.42)	−2.35 (−0.44)
WEIMuS (score)	22.9 ± 14.7 26.5 (12.5–32.8)	18.6 ± 12.8 17.0 (9.5–27.3)	+18.7	20.6 ± 14.9 18.5 (8.8–32.3)	+10.3	1.53	−1.26 (−0.24)	−0.74 (−0.14)
mental fatigue (score)	9.2 ± 7.8 7.0 (4.8–12.3)	8.5 ± 6.9 7.5 (2.8–13.5)	+7.8	9.5 ± 9.0 (1.5–14.0)	−3.1	0.41	−0.77 (−0.14)	−0.45 (−0.08)
physical fatigue (score)	13.7 ± 9.2 17.5 (4.8–22.0)	10.1 ± 7.0 10.0 (4.8–13.3)	+26.0	11.1 ± 7.8 9.5 (4.5–20.0)	+19.3	2.00	−1.56 (−0.29)	−1.91 (−0.36)
SSA (score)	4.7 ± 1.2 4.9 (3.4–5.6)	4.5 ± 1.2 4.5 (3.6–4.9)	−4.4	4.9 ± 1.1 4.9 (3.7–5.2)	+4.0	2.26	−0.31 (−0.06)	−0.97 (−0.18)

*Note*. Positive percentage values indicate improvements and negative percentage values indicate declines; *X*^2^*-*scores were taken from results of Friedman test; *Z*-scores were taken from results of Wilcoxon Signed Rank test. Effect size* r* was computed using formula *r* = *z*/square root of *N*; *∗* denotes statistical significance (*p* ≤ 0.05) for exact *p* values of Friedman test (two-tailed) or exact *p* value of Wilcoxon Signed Rank test (two-tailed) with Bonferroni adjustment for three measurements; EG-all: all participants receiving ePEP (EG-I + EG-W); *T*0/*T*0′: baseline measurements; *T*1/*T*1′: measurements after six-week ePEP; *T*2/*T*2′: measurements after 12-week self-regulated training period; SD: standard deviation; IQR: interquartile range (25th–75th); TUG: Timed-Up-and-Go-Test; 6MWT: Six-Minute-Walk-Test; FGA: Functional Gait Assessment; MusiQol: Multiple Sclerosis International Questionnaire of Quality of Life; WEIMuS: German MS-specific fatigue questionnaire—lower values indicate fatigue improvement; SSA: scale for self-efficacy towards physical exercise.

**Table 5:** Quasi-experimental design outcomes in walking ability, QoL, fatigue, and self-efficacy for EG-all in baseline (*T*0/*T*0′) and one year after baseline (*T*3).

Variable	EG-all (*n* = 8)				
	*T*0/*T*0′ mean ± SD median (IQR)	*T*3 mean ± SD median (IQR)	Percentage change *T*0-*T*1	*Z*-score	Effect size *r*
TUG (s)	7.6 ± 1.4 7.7 (6.3–8.7)	6.8 ± 1.5 6.7 (5.6–7.3)	+11.1	−2.38^*∗*^	−0.60
6MWT (m)	494.8 ± 129.2 461.2 (390.8–569.8)	513.8 ± 115.9 500.2 (468.4–580.8)	+3.8	−0.84	−0.21
FGA (score)	22.0 ± 6.7 23.0 (15.7–27.5)	23.8 ± 4.3 24.5 (23.0–26.8)	+8.0	−0.84	−0.21
MusiQol (score)	64.4 ± 15.4 67.7 (51.9–79.4)	69.0 ± 14.2 67.9 (56.2–82.9)	+7.1	−1.88	−0.42
WEIMuS (score)	19.8 ± 10.5 21.5 (14.0–27.8)	14.6 ± 7.0 14.5 (9.0–20.8)	+26.0	−1.36	−0.34
Mental fatigue (score)	6.6 ± 3.2 7.0 (5.25–9.0)	4.9 ± 3.0 4.0 (0.8–7.0)	+35.9	−1.36	−0.34
Physical fatigue (score)	13.1 ± 9.1 13.5 (5.5–22.0)	10.4 ± 6.6 7.5 (5.0–18.0)	+20.6	−1.05	−0.26
SSA (score)	4.9 ± 1.4 5.1 (5.3–9.0)	4.3 ± 0.8 4.6 (3.7–5.0)	−6.7	−1.18	−0.30

*Note*. Positive percentage values indicate improvements and negative percentage values indicate declines; *Z*-scores were taken from results of Wilcoxon Signed Rank test. Effect size* r* was computed using formula *r* = *z*/square root of *N*; *∗* denotes statistical significance (*p* ≤ 0.05) for exact *p* value of Wilcoxon Signed Rank test (two-tailed); EG-all: all participants receiving ePEP (EG-I + EG-W); *T*0/*T*0′: baseline measurements; *T*3: measurements one year after baseline; SD: standard deviation; IQR: interquartile range (25th–75th); TUG: Timed-Up-and-Go-Test; 6MWT: Six-Minute-Walk-Test; FGA: Functional Gait Assessment; MusiQol: Multiple Sclerosis International Questionnaire of Quality of Life; WEIMuS: German MS-specific fatigue questionnaire—lower values indicate fatigue improvement; SSA: scale for self-efficacy towards physical exercise.
